# Exploring strategies to optimise outcomes in hepatitis-associated aplastic anaemia patients following haematopoietic stem cell transplantation

**DOI:** 10.1038/s41598-024-55843-7

**Published:** 2024-03-02

**Authors:** Jia Li, Yilin Liu, Jieru Wang, Yan Wang, Aiming Pang, Donglin Yang, Xin Chen, Rongli Zhang, Jialin Wei, Qiaoling Ma, Weihua Zhai, Yi He, Erlie Jiang, Mingzhe Han, Sizhou Feng

**Affiliations:** 1 Haematopoietic Stem Cell Transplantation Center, State Key Laboratory of Experimental Haematology, National Clinical Research Center for Blood Diseases, Haihe Laboratory of Cell Ecosystem, Institute of Haematology and Blood Diseases Hospital, Chinese Academy of Medical Sciences and Peking Union Medical College, Tianjin, 300020 China; 2 Tianjin Institutes of Health Science, Tianjin, 301600 China; 3 Department of Haematology, The Affiliated Yantai Yuhuangding Hospital of Qingdao University, Yantai, China

**Keywords:** Haematopoietic stem cell transplant, Hepatitis-associated aplastic anaemia, Survival, Graft vs. host disease, Risk factors, Cancer, Diseases, Medical research, Oncology, Risk factors, Signs and symptoms

## Abstract

This study aimed to assess haematopoietic stem cell transplantation (HSCT) safety and efficacy while exploring strategies for optimising outcomes in patients with hepatitis-associated aplastic anaemia (HAAA). We retrospectively reviewed 35 HAAA patients who underwent HSCT at a large Chinese blood disease hospital between 2008 and 2022. HAAA patients receiving HSCT typically presented with severe (28.6%) and very severe (65.7%) AA. Male patients predominated (68.6%), with a median onset age of 23 years (range, 9–44). Haploidentical donor-HSCT and matched sibling donor-HSCT were in comparable proportions. The 5-year overall survival (OS) rate was 74.0%, with cumulative incidences of grade II–IV acute and chronic graft-versus-host disease (GVHD) at 37.1% and 22.4%, respectively. A diagnosis-to-HSCT interval ≥ 75 days, acute GVHD, and post-HSCT liver events (e.g., hepatic GVHD and a three-fold increase in aminotransferase or bilirubin) significantly worsened 5-year OS. In the multivariate models, recipients with sex-matched grafts had better OS, and those with younger male donors had a lower incidence of II–IV aGVHD. Higher HLA matching degree (HLA > = 7/10) was an independent prognostic factor associated with better OS and GFFS. A diagnosis-to-HSCT interval ≥ 75 days was predictive of post-transplant liver events in HAAA patients. In conclusion, HSCT was a safe and effective treatment for HAAA. Early transplantation, careful donor selection and improving post-transplant liver events were crucial to optimise outcomes.

## Introduction

Hepatitis-associated aplastic anaemia (HAAA) was initially reported in 1955 by Lorenz and Quaiser^1^. It is a variant of aplastic anaemia (AA) in which bone marrow failure follows an acute attack of hepatitis. Its pathogenesis remains poorly understood. Previous studies^2–4^ have shown that HAAA is more prevalent in adolescent males, and most patients do not exhibit evidence of active hepatitis A, B, C, D, E, G and Torque Teno virus during diagnosis. Both HAAA and non-viral hepatitis may share a common etiology, which is mainly T-cell mediated immune dysregulation.

Limited studies have investigated the outcomes after transplantation in a large cohort of HAAA patients. It was observed that 17 patients with AA in association with viral hepatitis who received matched sibling donor-haematopoietic stem cell transplantation (MSD-HSCT) had relatively satisfying post-transplant outcomes in 1984^5^. A recent study^6^ in China showed a 100% 3-year OS rate for 15 HAAA patients receiving haploidentical donor (HID)-HSCT. Analyzing risk factors for post-transplant outcomes can provide valuable evidence for treatment timing and donor selection in patients. Only one study conducted in 2010^2^ indicated that a younger age of 20 years or older (relative risk [RR]: 1.5), an interval of more than 140 days between diagnosis and HSCT (RR: 1.8), peripheral blood (PB) transplants instead of bone marrow (BM) transplants (RR for BM: 0.84) were predictors of survival for HAAA patients. It suggests that HSCT is a viable treatment option for HAAA patients. However, further research is needed to optimise outcomes.

To clarify these issues, our study reviewed the clinical characteristics and transplantation details of HAAA patients. We firstly investigated the post-transplant outcomes of these patients from multiple dimensions, such as infection, engraftment, graft-versus-host disease (GVHD), transplant-related mortality (TRM), and survival. Additionally, we performed univariate and multivariate analyses to identify risk factors for OS, GVHD-free, failure-free survival (GFFS), post-HSCT liver events, grade II–IV acute GVHD, and cytomegalovirus (CMV) viremia. This study provides valuable insight into the outcomes of HAAA patients and helps identify risk factors for improved clinical decision making.

## Patients and methods

### Patients

We conducted a retrospective analysis of 35 consecutive patients who underwent HSCT for HAAA between September 2008 and August 2022 at a 766-bed tertiary blood disease hospital in Tianjin, China. AA was diagnosed according to the Camitta criteria and patients with paroxysmal nocturnal haemoglobinuria (PNH) clones were included in the study, while those with hereditary bone marrow failure syndromes and myelodysplastic syndrome were excluded^7^. HAAA was defined as a specific subtype of AA, characterized by onset of pancytopenia with a hypoplastic bone marrow that traditionally occurs within 6 months of an elevation in serum aminotransferases^2,8^. Serological and virological testing confirmed negative hepatitis A, B, and C status. The study was approved by the Ethics Committee of the Institute of Haematology and Blood Diseases Hospital, and informed written consent was obtained from all patients or their guardians according to the Declaration of Helsinki.

### Procedures of transplantation

All patients were admitted to the laminar flow ward during HSCT until neutrophil engraftment. The conditioning regimen included a combination of anti-thymocyte globulin (ATG) (rabbit, 2.5 mg/kg/d × 5d; porcine, 20–25 mg/kg/d × 5d), cyclophosphamide (Cy) (40 or 50 mg/kg/d × 3d), fludarabine (Flu) (30 mg/m^2^/d, day − 5 to day − 1), with or without busulfan (Bu) (3.2 mg/kg/d × 2d). The GVHD prophylaxis regimen included the calcineurin inhibitors (either cyclosporine [CsA] or tacrolimus [FK506]), methotrexate (MTX), with or without mycophenolate mofetil (MMF).Additionally, granulocyte colony-stimulating factor (G-CSF) was administered to all patients from day + 6 until absolute neutrophil count (ANC) normalisation.

### Infection prevention strategies

Before transplantation, all patients received Paediatric Compound Sulfamethoxazole Tablets (1 g, twice daily for one week) to prevent Pneumocystis jirovecii pneumonia, alongside ganciclovir intravenously at a dosage of 10 mg/kg/day for 1 week to prevent CMV infection. For those without a pre-transplant invasive fungal disease (IFD) diagnosis, fluconazole was administered as a prophylactic treatment up to 3 months post-transplantation. Those diagnosed with IFD before transplantation were treated individually with itraconazole, voriconazole, micafungin, or caspofungin, depending on their specific pre-transplant conditions. Additionally, routine prophylactic treatment included antibiotics such as compound sulfamethoxazole and albendazole before transplantation.

### Definitions

Neutrophil engraftment was defined as the first occurrence of three consecutive days with an ANC ≥ 0.5 × 10^9^/L without G-CSF, while platelet engraftment was defined as the day when the platelet count reached ≥ 20 × 10^9^/L without transfusion for a week. Using short tandem repeat PCR, cells > 95% indicate complete donor chimerism, reflecting total donor-derived haematopoiesis. Donor cells between 5.0 and 95% suggest mixed chimerism. Levels under 5% represent recipient-exclusive haematopoiesis, indicating absent donor chimerism^9^. Primary graft failure (GF) was defined as not achieving neutrophil engraftment by day + 28, while patients with initial engraftment who later experienced recurrent pancytopenia with obviously hypocellular BM were considered to have secondary GF. CMV viremia was characterized as a positive detection in blood samples, identified through reverse transcriptase PCR, with a threshold of 1 × 10^3^ copies/mL. Post-transplant liver events were defined as veno-occlusive disease (VOD), viral hepatitis, hepatic GVHD, and a three-fold increase in alanine aminotransferase (ALT), aspartate aminotransferase (AST) or total bilirubin (TBIL) within 6 months post-HSCT. aGVHD was identified through its clinical features and graded according to the MAGIC criteria^10^. For cGVHD, the diagnosis and grading were based on the 2014 National Institutes of Health consensus^11^. TRM was defined as the incidence of death without disease progression. Death from non-transplant causes was considered a competing risk for TRM, and death from any cause was a competing risk for engraftment and GVHD. OS was defined as the time from HSCT to death from any cause or last follow-up. FFS was defined as survival with treatment response, where death, GF, and relapse were considered treatment failures. GFFS was defined as survival without grade III–IV acute GVHD, moderate to severe chronic GVHD, and treatment failures as described above. The normal range used for the primary analysis were as follows: 0–50 U/L for ALT, 0–50 U/L for AST, 5–21 μmol/L for TBIL, 0–3.4 μmol/L for direct bilirubin (DBIL), 23.9–336.2 ng/mL for serum ferritin, 56–86% for the proportion of CD3+ T cells, 33–58% for the proportion of CD4+ T cells, 13–39% for the proportion of CD8+ T cells, 5–22% for the proportion of CD19+ B cells, and 5–26% for the proportion of CD3−CD16/CD56+ NK cells to lymphocytes in PB.

### Statistical analysis

Statistical analysis was performed using SPSS software version 26.0 and R software package version 4.2.2. Continuous variables, such as CBC at diagnosis, interval from diagnosis to allo-HSCT, and cell compositions in allografts, were transformed into categorical variables using the cut-off value determined by R software. Kaplan–Meier survival analysis with log-rank test was used to estimate the probabilities of OS, FFS, and GFFS. The cumulative incidences of engraftment, CMV or EBV, GVHD, liver events and TRM were estimated using a competing risk model with Gray's test. A P-value of less than 0.05 was considered statistically significant. Finally, we included three factors as time-dependent variables in a multivariate Cox model or a competing risk model.

### Ethics approval

The study design was approved by the Ethics Committee of the Blood Diseases Hospital, Chinese Academy of Medical Sciences. All methods were performed in accordance with the relevant guidelines and regulations. Lot number: IIT2021011-EC-1. Please refer to the attached file.

### Patient consent

We have obtained written informed consent from the patient or patient’s parent/guardian.

## Results

### Basic characteristics and transplantation details

Table 1 summarizes the clinical characteristics of 35 patients who underwent allo-HSCT for HAAA over the past 15 years, with a median follow-up time of 1156 days (range, 130–5099) for survivors. Our study revealed that HAAA patients who received HSCT were more male (n = 24, 68.6%), with a median onset age of 23 years (range, 9–44), and only one patient was over 40 years old. The majority of HAAA cases were VSAA (n = 23, 65.7%) or SAA (n = 10, 28.6%). Mucocutaneous hemorrhage was the primary symptom in 60.0% of HAAA patients, followed by infections with or without fever (37.1%), while 17.1% of patients presented with fatigue only. The median time from hepatitis to diagnosis was 60 days (range, 0–210). For patients with available medical information at the onset of hepatitis, over half had AST > 10 × upper limit of normal (ULN) (14/23), ALT > 20 × ULN (17/25), TBIL > 5 × ULN (11/19), and DBIL > 10 × ULN (13/17). Through meticulous serologic and virologic testing, we established that patients in our study were not affected by hepatitis A, B, or C. Additionally, we rigorously evaluated and subsequently excluded the possibility of drug-induced hepatitis in all cases. The majority of patients had high serum ferroprotein levels at HAAA diagnosis, with a median value of 567.85 (interquartile range [IQR], 299.43–905.40) ng/mL. Only 9.45% (n = 3) of the 32 evaluable patients had PNH clones.

**Table 1:** Clinical characteristics of patients with hepatitis‑associated aplastic anaemia (HAAA).

Characteristics	HAAA n = 35
Transplant year	
2008–2016	16 (45.7%)
2017–2022	19 (54.3%)
Gender	
Male	24 (68.6%)
Female	11 (31.4%)
Patient age at diagnosis, years, median (range)	23 (9–44)
Patient age at diagnosis, years	
9–19	15 (42.9%)
20–29	11 (31.4%)
30–39	8 (22.9%)
40–	1 (2.9%)
Patient age at transplantation, years, median (range)	23 (9–44)
Diagnosis	
SAA	10 (28.6%)
VSAA	23 (65.7%)
NSAA	2 (5.7%)
CBC at diagnosis	
White blood cells, 10^9^/L	0.97 (0.45–1.70)
Neutrophils, 10^9^/L	0.1550 (0.0200–0.4100)
Hemoglobin, g/L	77 (68–86)
Platelets, 10^9^/L	10 (6–16)
Reticulocytes, 10^12^/L	0.0093 (0.0050–0.01515)
Serum ferroprotein at diagnosis, ng/mL	567.85 (299.43–905.40)
Liver function at diagnosis of hepatitis	
ALT, IU/L	1282.00 (757.50–1765.00)
AST, IU/L	847.50 (353.63–1151.25)
TBIL, μmol/L	131.50 (31.70–234.00)
DBIL, μmol/L	114.15 (47.18–153.68)
Interval from hepatitis to diagnosis, days, median (range)	60 (0–210)
PNH clone	3 (9.4%)^a^
Primary symptoms	
Mucocutaneous hemorrhage	21 (60.0%)
Infections with or without fever	13 (37.1%)
Fatigue only	6 (17.1%)
Lymphocyte subsets in peripheral blood at diagnosis	
CD3+ cells, %	78.10 (66.88–82.00)
CD3+ cells, /μL	562 (206–752)
CD3+CD4+ cell, %	26.00 (11.00–44.00)
CD3+CD4+ cell, /μL	136 (29–383)
CD3+CD8+ T cells, %	33.20 (28.50–46.00)
CD3+CD8+ T cells, /μL	224 (122–423)
CD4/CD8 ratio	0.77 (0.25–10.32)
CD19+ B cells, %	13.85 (7.44–23.75)
CD19+ B cells, /μL	104 (22–214)
CD3−CD16/CD56+ NK cells, %	7.35 (2.80–12.00)
CD3−CD16/CD56+ NK cells, /μL	47 (14–100)
Survivor follow-up time, days, median (range)	1156 (130–5099)

Categorical variables are presented as numbers (percentiles); continuous variables are presented as median (interquartile range) unless otherwise stated.

*ALT* alanine aminotransferase, *AST* aspartate aminotransferase, *BM* bone marrow, *Bu* busulfan, *CBC* complete blood count, *DBIL* direct bilirubin, *HAAA* hepatitis‐associated aplastic anaemia, *NSAA* non- severe aplastic anaemia, *PB* peripheral blood, *PNH* paroxysmal nocturnal hemoglobinuria, *SAA* severe aplastic anaemia, *TBIL* total bilirubin, *VSAA* very severe aplastic anaemia.

**Figure 1:**
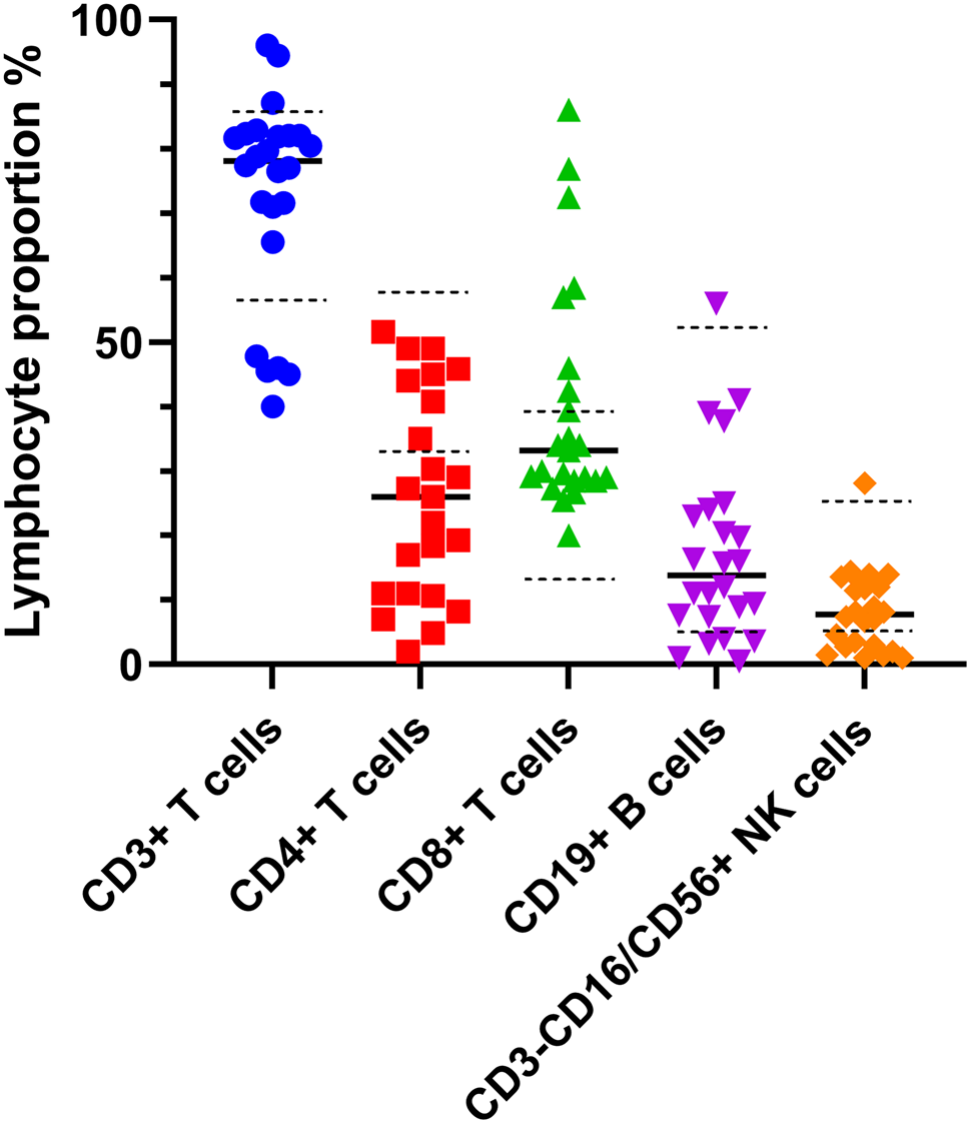
Proportion of peripheral blood lymphocyte subsets in HAAA patients before treatment. The median proportion of each lymphocyte subset to lymphocytes is shown with a solid black line, and the normal reference range for lymphocyte subsets is represented with a dashed black line.

Moreover, lymphocyte proportions are displayed in Fig. 1. Of the 23 patients with pre-treatment lymphocyte subset data, more than half of the HAAA patients (56.5%, n = 13) had a CD4 cell proportion below the normal level prior to treatment, and 1/3 of the patients (34.8%, n = 8) had a CD3−CD16/CD56+ T cell proportion below normal, suggesting that the PB lymphocyte imbalance plays a central role in the immune-mediated pathogenesis of HAAA.

Additionally, we found that the median time from diagnosis to HSCT in HAAA patients was 75 days (range, 34–412) (Table 2). Prior to HSCT, two patients received immunosuppressive therapy based on ATG. None of the HAAA patients underwent matched unrelated donor (MUD)-HSCT, while haploidentical donor (HID)-HSCT (n = 18) and MSD-HSCT (n = 17) were used in similar proportions. The degree of donor–recipient blood type and sex matching was unevenly distributed. All patients had a haematopoietic cell transplantation specific comorbidity index of 0–1. The vast majority of HAAA patients received GVHD prophylaxis with CsA + MTX ± MMF (84.4%) and conditioning regimen with Cy + Flu + ATG (82.9%). PB was the primary source of stem cells for HAAA patients (82.9%, n = 29). The median mononuclear cell count (MNC) was 10.00 × 10^8^/kg of recipient weight, and the median CD34+ cell count was 2.92 × 10^6^/kg of recipient weight, respectively.

**Table 2:** Transplantation details of patients with hepatitis‑associated aplastic anaemia (HAAA).

Characteristics	HAAA n = 35
Interval from diagnosis to HSCT, days, median (range)	75 (34–412)
IST before HSCT	
ATG-based	2 (5.7%)
CsA-based	22 (62.9%)
No	11 (31.4%)
HCT-CI pre-HSCT	
0–1	32 (100.0%)^a^
≥ 2	0 (0.0%)^a^
Graft type	
PB only	29 (82.9%)
PB + BM	5 (14.3%)
BM only	1 (2.9%)
Blood type	
Match	24 (68.6%)
Major mismatch	2 (5.7%)
Minor mismatch	5 (14.3%)
Mismatch	4 (11.4%)
Donor–patient sex match	
Female to female	6 (17.1%)
Female to male	7 (20.0%)
Male to male	16 (45.7%)
Male to female	6 (17.1%)
HLA type	
5/10	10 (31.3%)^a^
6/10	1 (3.1%)^a^
7/10	3 (9.4%)^a^
8/10	2 (6.3%)^a^
9/10	0 (0.0%)^a^
10/10	16 (50.0%)^a^
Conditioning regimen	
Bu + Cy + Flu + ATG	6 (17.1%)
Cy + Flu + ATG	29 (82.9%)
GVHD prophylaxis	
CsA + MTX + MMF	12 (37.5%)
FK506 + MTX + MMF	2 (6.3%)
CsA + MTX	15 (46.9%)
FK506 + MTX	3 (9.4%)
Cell compositions in allografts	
MNC, 10^8^/kg	10.00 (8.02–11.90)
CD34+ cells, 10^6^/kg	2.92 (2.29–4.16)
CD3+ T cells, 10^6^/kg	169.17 (109.67–230.26)
CD3+ CD4+ T cells, 10^6^/kg	86.57 (56.60–123.30)
CD3+ CD8+ T cells, 10^6^/kg	64.94 (41.39–105.77)
CD19+ B cells, 10^6^/kg	38.52 (17.08–66.63)

Categorical variables are presented as numbers (percentiles); continuous variables are presented as median (interquartile range) unless otherwise stated.

*ATG* anti‐thymocyte globulin, *BM* bone marrow, *Bu* busulfan, *CBC* complete blood count, *CsA* cyclosporin, *Cy* cyclophosphamide, *FK506* tacrolimus, *Flu* fludarabine, *GVHD* graft‐versus‐host disease, *HAAA* hepatitis‐associated aplastic anaemia, *HCT‐CI* haematopoietic cell transplantation specific comorbidity index, *HID* haploidentical donor, *HLA* human leukocyte antigen, *IST* immunosuppressive therapy, *MMF* mycophenolate mofetil, *MNC* mononuclear cells, *MSD* matched sibling donor, *MTX* methotrexate, *MUD* matched unrelated donor, *PB* peripheral blood.

### Outcomes

#### Engraftment and infection

We analyzed the post-transplant outcomes of HAAA patients in terms of engraftment, infection, GVHD, and survival (Table S1). All patients achieved neutrophil engraftment with the median engraftment time of 12 days (range, 10–20) (Fig. S1A). Platelet engraftment occurred in a median of 14 days (range, 8–45) with a 30-day cumulative incidence of 88.6% (95% CI: 75.8–96.4%) (Fig. S1B). Secondary GF occurred in only one patient at day 90 after HSCT. Additionally, 17.1% (n = 6) of HAAA patients suffered from bacteremia, and 37.1% (n = 13) suffered from severe pneumonia within 100 days after transplantation. Early CMV viremia within 100 days after HSCT occurred in 40.9% (95% CI: 26.6–59.1%; n = 14) of patients, while early EBV viremia occurred in only three patients (Fig. S1C,D). The median interval of early CMV viremia was 42 days (range, 21–64). In contrast to EBV viremia (33.3% vs. 77.7%, P = 0.015), CMV viremia did not have a significant impact on survival (57.1% vs. 84.6%, P = 0.176) (Table 3).

**Table 3:** Univariate and multivariate analyses of adverse factors associated with OS and GFFS.

Outcome	No. of events/evaluable (5-year survival rate %)	Univariate	Multivariate	
		P value (log rank)	P value (COX)	Hazard ratio (95% confidence interval)
OS				
Interval from diagnosis to HSCT, days		0.001*		
< = 75	0/18 (100.0%)			
> 75	8/17 (46.6%)			
Degree of HLA matching		0.033*^#^	***0.006***	8.2 (2.6–19.7)
< 7/10	5/12 (44.4%)			
> = 7/10	3/23 (86.1%)			
Donor age, years		***0.096***		
< 20	0/9 (100.0%)			
> = 20	8/25 (65.8%)			
Donor–patient sex match		***0.057***^***#***^	***0.008***	7.1 (2.4–16.5)
Sex match	3/22 (81.4%)			
Sex mismatch	5/13 (59.2%)			
EBV viremia after HSCT		0.015*		
Yes	2/3 (33.3%)			
No	6/32 (77.7%)			
Liver events after HSCT		0.035*^#^	0.110	
Yes	6/14 (51.4%)			
No	2/21 (90.2%)			
II–IV aGVHD after HSCT		< 0.001*		
Yes	7/13 (35.2%)			
No	0/21 (100.0%)			
III–IV aGVHD after HSCT		< 0.001*		
Yes	3/4 (25.0%)			
No	4/30 (83.4%)			
CD34+ cells in allografts, 10^6^/kg		0.014*		
< = 4.5	4/27 (81.8%)			
> 4.5	3/5 (40.0%)			
CD3+ CD4+ cells in allografts, 10^6^/kg		0.002*		
< = 125.0	2/23 (91.3%)			
> 125.0	4/6 (25.0%)			
CD3+ CD8+ cells in allografts, 10^6^/kg		***0.075***		
< = 52.0	1/13 (92.3%)			
> 52.0	5/16 (49.2%)			
GFFS				
Interval from diagnosis to HSCT, days		0.036*^#^	0.049*	3.8 (1.0–14.6)
< = 75	3/18 (82.2%)			
> 75	8/17 (47.6%)			
Degree of HLA matching		0.004*^#^	0.019*	4.4 (1.3–15.5)
< 7/10	7/12 (35.0%)			
> = 7/10	4/23 (81.0%)			
MNC in allografts, 10^8^/kg		0.028*		
< = 12.5	7/26 (70.2%)			
> 12.5	4/6 (33.3%)			
CD3+ cells in allografts, 10^6^/kg		0.044*^#^	0.112	
< = 115.0	1/9 (88.9%)			
> 115.0	10/20 (48.0%)			

A P value in italics and bold means < 0.1, followed by * means < 0.05, followed by # means that this variable was included in the multifactorial analysis.

*aGVHD* acute graft‐versus‐host disease, *GFFS* graft‐versus‐host disease‐free and failure‐free survival, *HLA* human leukocyte antigen, *HSCT* haematopoietic stem‐cell transplantation, *OS* overall survival, *Ret* reticulocytes, *MNC* mononuclear cells.

#### Liver events

Our study focused on post-transplant liver events in HAAA patients. We found that 40% of the patients (14 in total) experienced hepatic events. We found that 40% of the patients (14 in total) experienced hepatic events after transplantation. Among these, eight patients developed hepatic GVHD, while the remaining 6 experienced significant enzyme elevation due to various other causes. None suffered from VOD and viral hepatitis. The 5-year OS rate was significantly lower for patients who developed post-HSCT liver events (51.4% [95% CI: 26.9–82.8%] vs. 90.2% [95% CI: 83.0–100.0%], P = 0.035) (Fig. 3A).

#### GVHD and survival

**Figure 2:**
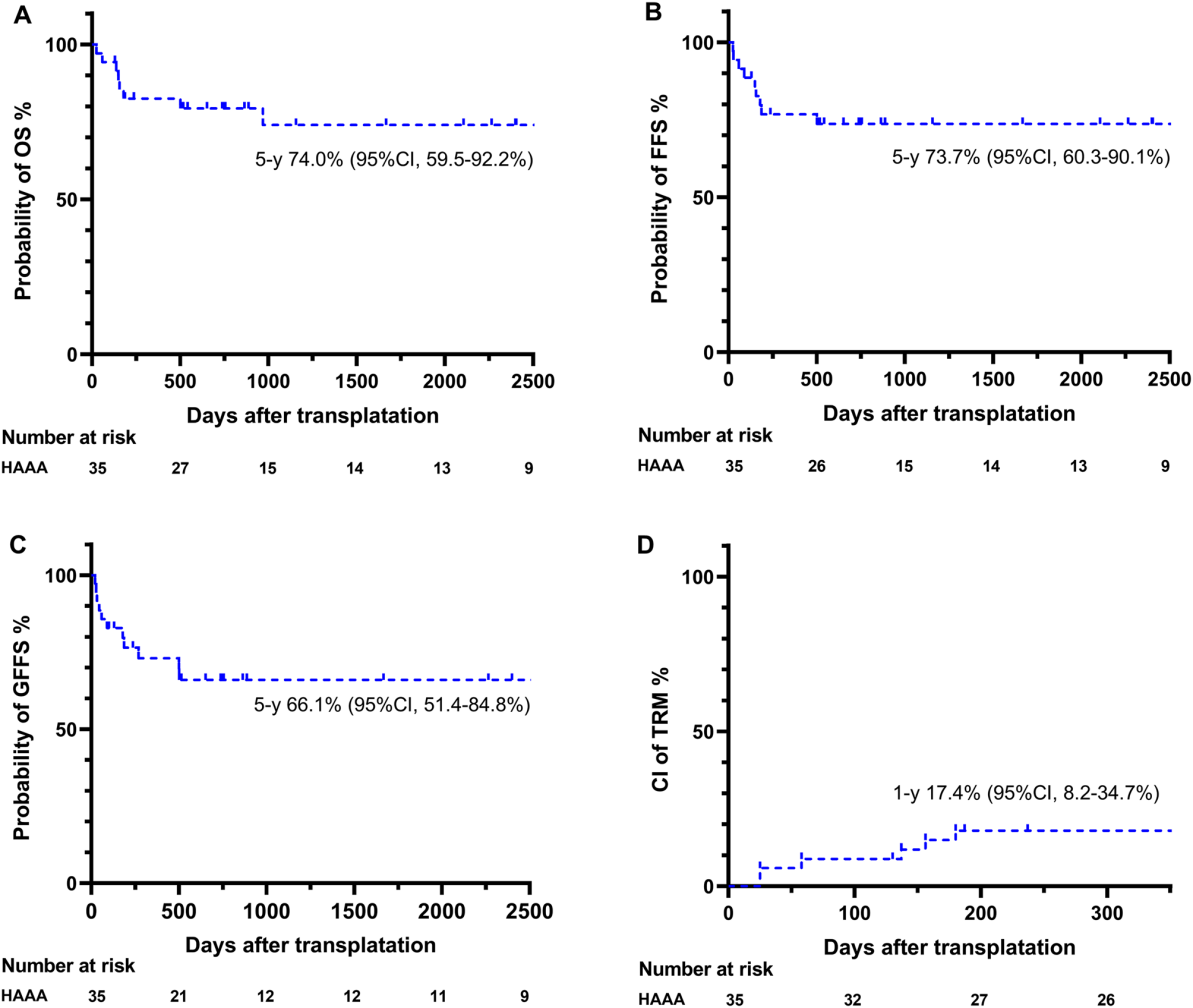
Survival outcomes in HAAA patients after HSCT. (**A**) Overall survival (OS), (**B**) failure-free survival (FFS), (**C**) GVHD-free, failure-free survival (GFFS), and (**D**) transplantation-related mortality (TRM) are shown. *CI* cumulative incidence, *HAAA* hepatitis-associated aplastic anaemia, *GVHD* graft-versus-host disease.

Of the 35 patients with HAAA, 37.1% (95% CI: 23.5–55.2%; n = 13) developed grade II–IV aGVHD at 100 days post-HSCT, while 11.4% (95% CI: 4.5–27.6%; n = 4) developed grade III–IV aGVHD within the same period (Fig. S2A,B). The cumulative incidence of mild to severe cGVHD and moderate to severe cGVHD at 5 years was 22.4% (95% CI: 8.7–36.6%; n = 6) and 16.0% (95% CI: 4.8–29.5%; n = 4), respectively (Fig. S2C,D). Among 35 patients, 3 died from III–IV aGVHD, 3 from severe pneumonia, 1 from sepsis, and 1 from an accident within 5 years after transplant. The estimated 5-year rates for OS, FFS, and GFFS were 74.0% (95% CI: 59.5–92.2%), 73.7% (95% CI: 60.3–90.1%), and 66.1% (51.4–84.8%), respectively (Fig. 2A–C). Six patients (17.4%; 95% CI: 8.2–34.7%) died within 1 year due to transplant-related infection or aGVHD (Fig. 2D). In addition, grade II–IV aGVHD (35.2% vs. 100.0%, P < 0.001) and grade III–IV aGVHD (25.0% vs. 83.4%, P < 0.001) were significant risk factors that worsened OS (Table 3).

### Univariate and multivariate analysis

#### Survival

**Figure 3:**
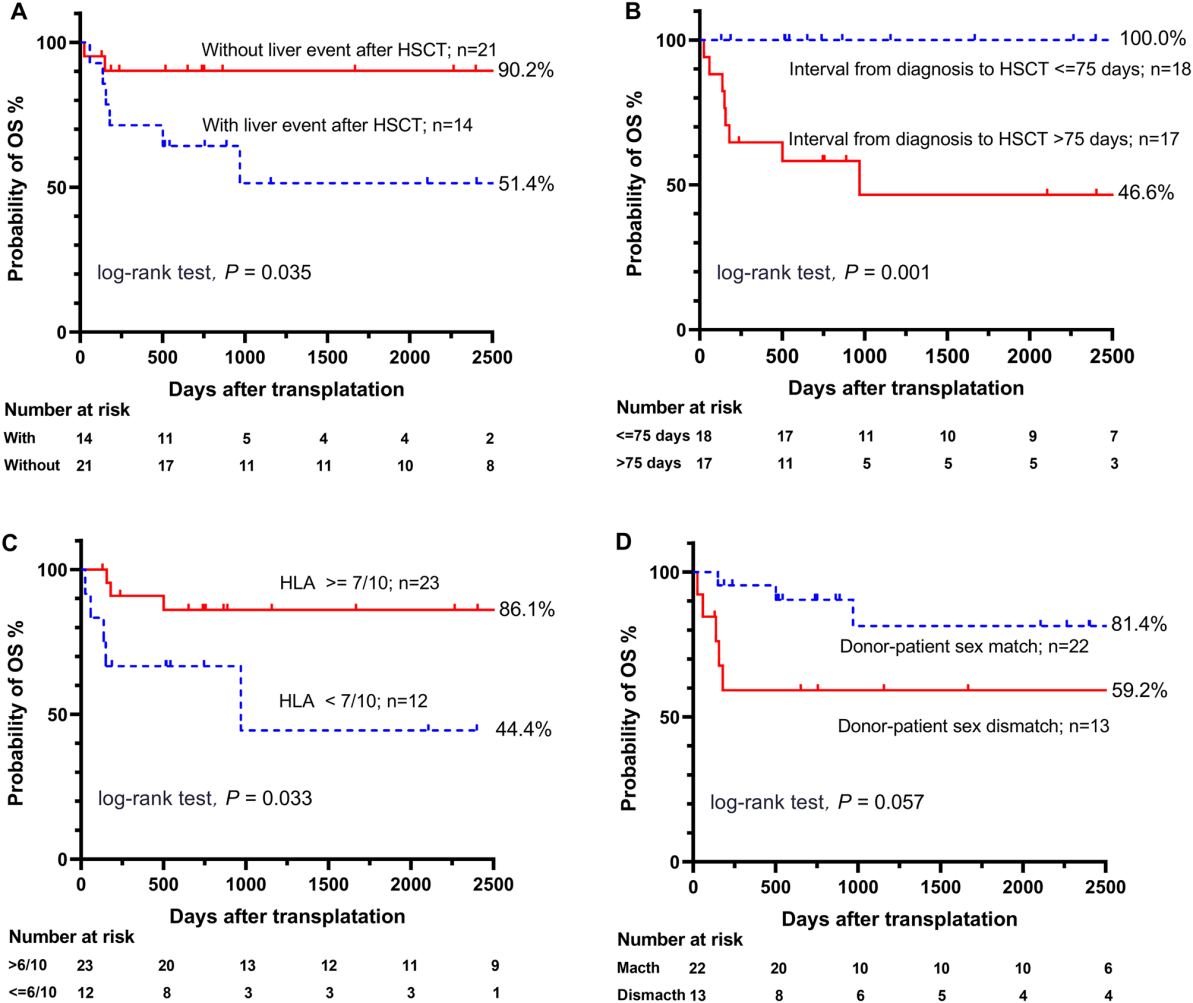
Overall survival (OS) of HAAA patients after transplantation, stratified by: (**A**) presence or absence of liver events after HSCT; (**B**) interval from diagnosis to HSCT (< = 75 days vs. > 75 days); (**C**) degree of HLA matching between recipient and donor (lower [< 7/10] vs. higher [> = 7/10]); (**D**) donor-patient sex-matching (matched vs. mismatched). *HAAA* hepatitis-associated aplastic anaemia, *HSCT* haematopoietic stem cell transplantation, *HLA* human leukocyte antigen.

**Figure 4:**
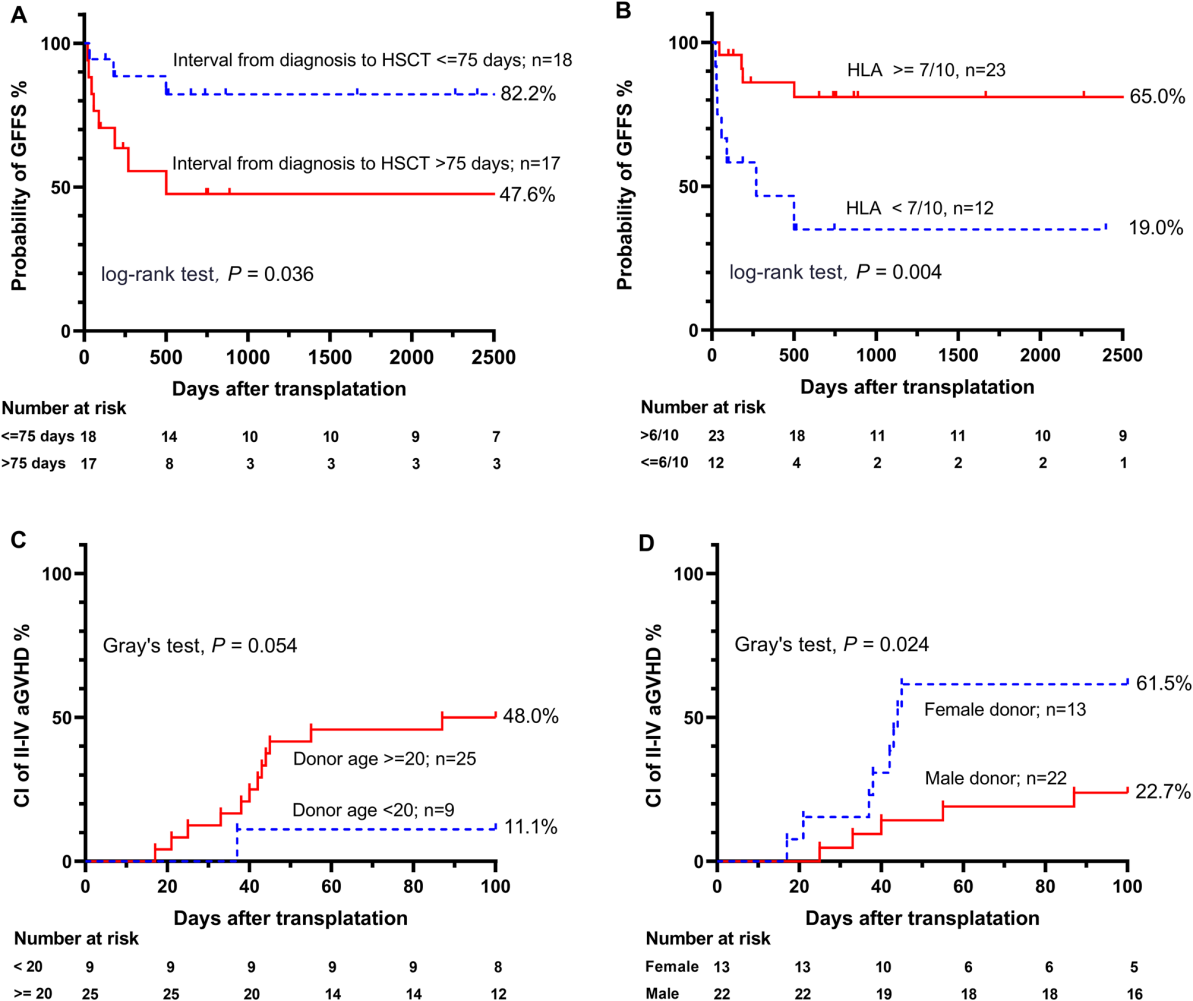
GVHD-free, failure-free survival (GFFS) of HAAA patients after transplantation, stratified by: (**A**) interval from diagnosis to HSCT (< = 75 days vs. > 75 days); (**B**) degree of HLA matching between recipient and donor (lower [< 7/10] vs. higher [> = 7/10]). Grade II–IV acute GVHD of HAAA patients after transplantation, stratified by: (**C**) donor age group (> = 20 years vs. < 20 years); (**D**) donor sex (female vs. male). *CI* cumulative incidence, *HAAA* hepatitis-associated aplastic anaemia, *GVHD* graft-versus-host disease, *HLA* human leukocyte antigen.

Patients with > 75 days between diagnosis and HSCT had lower OS (46.6% [95% CI: 22.6–74.4%] vs. 100.0%, P = 0.001) (Fig. 3B). The survivors' donors were marginally younger (< 20 years, 100.0% vs. 65.8%, P = 0.096) (Table 3). The analysis revealed that HSCT patients receiving grafts from donors with lower HLA matching (HLA < 7/10) had a 5-year estimated OS rate of 44.4% (95% CI: 18.2–80.2%), compared to 86.1% (95% CI: 72.7–100.0%) for those with higher HLA matching (P = 0.033) (Fig. 3C). Additionally, patients with sex-mismatched grafts showed a lower 5-year OS rate of 59.2% (95% CI: 37.1–94.5%), versus 81.4% (95% CI: 63.4–100.0%) in matched grafts (P = 0.057) (Fig. 3D). Patients with a shorter interval (< = 75 days) between diagnosis and HSCT had a better GFFS rate of 82.2% (95% CI: 65.8–100.0%) compared to 47.6% (95% CI: 27.6–82.2%) for longer intervals (P = 0.036) (Fig. 4A). Higher HLA matching (HLA > = 7/10) also correlated with improved GFFS rates: 81.0% (95% CI: 65.8–99.7%) versus 35.0% (95% CI: 14.8–82.8%) (P = 0.004) (Fig. 4B). The univariate analysis identified that higher counts of MNC, CD34+ cells, CD3+ T cells, CD4+ T cells, and CD8+ T cells in allografts, above the specified cut-off values, were associated with poorer OS or GFFS (Table 3).

Multivariate analysis showed that lower degrees of HLA-matched donors (HLA < 7/10) (Hazard ratio [HR]: 8.2, 95% CI: 2.6–19.7, P = 0.006) and donor-patient sex mismatch (HR: 7.1, 95% CI: 2.4–16.5, P = 0.008) were predictors of poor OS after HSCT. Interval from diagnosis to HSCT over 75 days (HR: 3.8, 95% CI: 1.0–14.6, P = 0.049) and lower degrees of HLA-matched donors (HR: 4.4, 95% CI: 1.3–15.5, P = 0.019) were independent risk factors for inferior GFFS.

#### Liver events

Given that post-transplant liver events significantly affected patient survival, we conducted an analysis to identify predictors (Table 4). In multifactorial analysis, an interval exceeding 75 days between HAAA diagnosis and HSCT emerged as a predictor of post-transplant liver events in HAAA patients (HR: 6.9, 95% CI: 1.8–27.0, P = 0.005).

**Table 4:** Univariate and multivariate analyses of adverse factors associated with II–IV aGVHD and CMV viremia.

Outcome	No. of events/evaluable (100-day cumulative incidence %)	Univariate	Multivariate	
		P value (fine gray)	P value (fine gray)	Hazard ratio (95% confidence interval)
Liver events after HSCT				
Interval from diagnosis to HSCT, days		0.004*^#^	0.005*	6.9 (1.8–27.0)
< = 75	3/18 (17.0%)			
> 75	11/17 (64.7%)			
HID group		0.363^*#*^	0.150	
HID	6/18 (33.3%)			
MSD	8/17 (47.7%)			
IST before HSCT		0.034*^#^	0.240	
ATG-based	2/2 (100.0%)			
CsA-based	5/22 (22.7%)			
No	7/11 (63.6%)			
II–IV aGVHD				
PLT at diagnosis, 10^12^/L		0.017*^#^	0.215	
< 10	9/15 (60.0%)			
> = 10	4/20 (20.0%)			
Donor age, years		***0.054***^***#***^	0.017*	6.8 (1.6–21.3)
< 20	1/9 (11.1%)			
> = 20	12/25 (48.0%)			
Donor sex		0.024*^#^	0.005*	5.6 (1.7–18.7)
Female donor	8/13 (61.5%)			
Male donor	5/22 (22.7%)			
CMV viremia				
Patient age at transplantation, years		***0.067***^***#***^	0.870	
9–19	7/15 (49.7%)			
20–29	1/10 (11.1%)			
30–39	6/9 (66.7%)			
40–	0/1 (0.0%)			
Donor type		0.010*^#^	0.002*	6.6 (2.0–21.2)
HID	11/18 (64.1%)			
MSD	3/17 (17.6%)			
Conditioning regimen		0.149^***#***^	0.333	
Bu + Cy + Flu + ATG	4/6 (66.7%)			
Cy + Flu + ATG	10/29 (35.5%)			

A P value in italics and bold means < 0.1, followed by * means < 0.05, followed by # means that this variable was included in the multifactorial analysis.

*aGVHD* acute graft‐versus‐host disease, *ATG* anti‐thymocyte globulin, *CsA* cyclosporin, *Bu* busulfan, *CMV* cytomegalovirus, *Cy* cyclophosphamide, *Flu* fludarabine, *HID* haploidentical donor, *MSD* matched sibling donor, *PLT* platelet.

#### GVHD and CMV viremia

In addition, the univariate analysis of the Fine-Gray model indicated that the older donor group had a marginally higher incidence of grade II–IV aGVHD (48.0% [95% CI: 30.7–68.8%] vs. 11.1% [95% CI: 1.6–56.7%], P = 0.054) (Fig. 4C). Similarly, patients who received HSCT from female donors also had a higher incidence of grade II–IV aGVHD (61.5% [95% CI: 37.2–86.0%] vs. 22.7% [95% CI: 10.2–46.3%], P = 0.024) (Fig. 4D). Moreover, the multivariate HR of the older donor group and female donor group also reached the significant levels of 6.8 (95% CI: 1.6–21.3, P = 0.017) and 5.6 (95% CI: 1.7–18.7, P = 0.005), respectively (Table 4). The multivariate competing risk mode further confirmed that HID-HSCT was a predictor for the occurrence of CMV viremia (HR: 6.6, 95% CI: 2.0–21.2, P = 0.002).

## Discussion

HAAA is a relatively rare condition, accounting for approximately 3–12% of newly diagnosed acquired AA cases^2,3,12^. The mean interval between onset of hepatitis and first indication of AA was 2 months, which was consistent with previous studies^13–15^. Markedly elevated aminotransferases and conjugated hyperbilirubinemia were observed at diagnosis of hepatitis preceding pancytopenia.

We investigated various aspects of post-transplant outcomes in patients with HAAA. In our research on HAAA patients, a substantial 65.7% had very severe aplastic anemia. They exhibited low initial platelet counts (median 10 × 10^9^/L) and marked anemia (HGB, median 77 g/L). This led to a high transfusion requirement, contributing to notably higher ferritin levels in these patients, indicative of potential iron overload issues. The majority of patients (94.3%) opted for transplantation over ATG-based immunosuppressive therapy. The 2010 report from the EBMT aplastic anaemia working party^2^ demonstrated a 10-year actuarial survival rate of 70% after transplantation, with most HAAA patients receiving MSD-HSCT. More recently, only one study conducted by the Japan Society for Haematopoietic Cell Transplantation^16^ reported a 5-year overall survival (OS) rate of 86.0% and a 100-day cumulative incidence of grade II–IV acute graft-versus-host disease (aGVHD) at 11.1% among HAAA patients who received HSCT from HLA-identical donors. In our published article^4^, we conducted a comparative analysis, matching 30 patients who underwent HAAA procedures at our center with patients without HAAA. The findings showed that estimated 5-year OS rate (75.8% vs. 86.5%, P = 0.409) and FFS rate (74.0% vs. 83.2%, P = 0.485) after HSCT were slightly lower but not statistically significantly different from those in the non-HAAA group. All HAAA patients achieved neutrophil engraftment and the 30-day cumulative incidence of platelet engraftment was 88.6%. Overall, HAAA patients had satisfactory outcomes after transplantation.

In this study, we first analysed the risk factors influencing survival, aGVHD, and early CMV viremia in patients with HAAA. Our findings emphasized the importance of early referral, with a 75-day cut-off providing optimal separation of patients receiving early and late HSCT, resulting in different OS rates (100% vs. 46.6%, P = 0.001). Our cut-off value was significantly shorter than the waiting period from diagnosis to transplantation in previous studies, which affects survival in idiopathic AA (> 12 months, HR: 2.18, P = 0.027)^17^ and all acquired AA (> 3 months, RR: 1.44, P = 0.031)^18^.

We placed great importance on post-transplant liver events within 6 months for HAAA patients. A study from China^6^ identified two liver events after HSCT in 15 HAAA patients who underwent HID-HSCT, and a study from Japan^16^ identified one case of VOD in 37 adult HAAA patients who underwent MSD or MUD-HSCT. In our study, 40% of patients (14 in total) experienced hepatic events post-transplantation. Among them, 8 were diagnosed with hepatic GVHD, while 6 others showed significant liver enzyme elevations from varied causes. Two patients improved after modifying potentially hepatotoxic drugs and receiving hepatoprotective therapy, indicating drug-related liver issues. One patient, despite initial improvement after stopping tigecycline and receiving hepatoprotective therapy, ultimately succumbed to complex complications, leaving the cause of liver dysfunction unresolved. Another patient's liver condition worsened due to CMV and EBV viremia, ultimately proving fatal. The remaining two patients showed improvement with hepatoprotective therapy alone, making the precise cause of their post-transplant hepatitis unclear. This data highlights the multifaceted nature of post-transplant liver events, encompassing factors like drug toxicity, viral infections, and GVHD. This study is the first to demonstrate that post-transplant liver events, which have not been identified as a risk factor in patients transplanted for idiopathic AA, have an adverse impact on the survival of patients with HAAA. Our findings emphasize the importance of the timely intervention in mitigating post-transplant liver events.

Our study also confirmed that HLA locus matching < 7/10 was an independent risk factor that significantly worsened OS (HR: 8.2, 95% CI: 2.6–19.7, P = 0.006) and GFFS (HR: 4.4, 95% CI: 1.3–15.5, P = 0.019) in HAAA patients. In addition, many studies^19–21^ have confirmed that AA patients who receive HID, MSD, or MUD-HSCT have similar 3-year OS and FFS rates, but the HID group still has a higher incidence of GVHD and early CMV viremia. We also found that HAAA patients were more likely to experience 100-day CMV viremia after HID-HSCT (64.1% vs. 17.6%, P = 0.010). The gender and age of the donors also affect the outcome of the transplant. As with recipients with AA^22^, aGVHD was more common and survival was marginally worse in the older donor group with HAAA. Additionally, a study involving 1481 patients who underwent HSCT for AA from 28 countries^23^ reported a significantly higher 5-year OS in the donor-patient sex-matched group (68% vs. 60%, P = 0.001), and male patients with female donors were at an increased risk of severe GVHD (RR: 1.33, P = 0.03). In our adjusted multivariate models, we also observed better survival in HAAA recipients with sex-matched grafts and a lower incidence of aGVHD in recipients with male donors. Therefore, a younger male donor may predict a better post-transplant outcome for HAAA patients, and transplantation from donors with a higher degree of HLA matching may result in improved survival rates.

Pursuing higher doses of certain cells in allografts may be counterproductive due to poorer survival. The optimal CD34+ cell count is uncertain and varies by graft type. Several studies^24–26^ supported that higher CD34+ progenitor doses improve OS, but others^27,28^ argued that excessively high CD34 cell counts may lead to elevated TRM (> 5 × 10^6^/kg) and extensive cGVHD morbidity (> 8 × 10^6^/kg). In addition, the correlation between CD3+, CD4+ and CD8+ doses in the graft and post-transplant outcomes remains controversial^25,29,30^. We found that in HAAA patients, higher doses of CD34+ (> 4.5 × 10^6^/kg), CD4+ (> 125.0 × 10^6^/kg) and CD8+ (> 52.0 × 10^6^/kg) resulted in poorer 5-year OS, and higher MNC (> 12.5 × 10^8^/kg) and CD3+ (> 115.0 × 10^6^/kg) doses were also associated with poorer 5-year GFFS.

The study's small sample size due to the low prevalence of HAAA limits the generalizability of the results. In particular, multivariate analysis of more cases is needed to confirm these findings. Additionally, the retrospective nature of the study meant that patient inclusion depended on physician judgment, which resulted in a limited ability to comprehensively assess patients.

In conclusion, allo-HSCT has been demonstrated to be a safe and effective treatment for HAAA. Optimal donors are young males with high HLA matching. To optimise outcomes, it is crucial to perform transplantation within 75 days and enhance post-transplant management of GVHD and liver events.

### Supplementary Information

Supplementary Information.

## Data Availability

The data that support the findings of this study are available from the corresponding author upon reasonable request.
