# Supplementary material for: Exploring strategies to optimise outcomes in hepatitis-associated aplastic anaemia patients following haematopoietic stem cell transplantation
Source: Sci Rep. 2024 Mar 2;14:5178. doi: 10.1038/s41598-024-55843-7 (PMC10908854; doi:10.1038/s41598-024-55843-7)
Supplement: Supplementary file 1 — Supplementary Information. [file 41598_2024_55843_MOESM1_ESM.docx]

| Table S1. Post-transplant outcomes of patients with HAAA. | |
| --- | --- |
| Characteristics | **HAAA**  **n=35** |
| Engraftment |  |
| *N*, days, median (range) | 12 (10-20) |
| PLT, days, median (range) | 14 (8-45) |
| CI of 30-day *N* engraftment | 100 |
| CI of 30-day PLT engraftment | 88.6 (75.8-96.4%) |
| Primary graft failure | 0 (0.0%) |
| Secondary graft failure | 1 (2.9%) |
| Infection within 100 days after HSCT |  |
| CI of 100-day CMV viremia | 40.9 (26.6-59.1%) |
| Interval from HSCT to CMV viremia, days, median (range) | 42 (21-64) |
| CI of 100-day EBV viremia | 8.6 (2.8-24.3%) |
| Interval from HSCT to EBV viremia, days, median (range) | 53 (40-96) |
| Bacteremia | 6 (17.1%) |
| Severe pneumonia | 13 (37.1%) |
| GVHD and Survival |  |
| 100-day II-IV° aGVHD | 37.1 (23.5-55.2%) |
| 100-day III-IV° aGVHD | 11.4 (4.5-27.6%) |
| 5-year mild to severe cGVHD | 22.4 (8.7-36.6%) |
| 5-year moderate to severe cGVHD | 16.0 (4.8-29.5%) |
| 1-year TRM | 17.4 (8.2-34.7%) |
| 5-year OS | 74.0 (59.5-92.2%) |
| 5-year FFS | 73.7 (60.3-90.1%) |
| 5-year GFFS | 66.1 (51.4-84.8%) |
| *Note*: Categorical variables are presented as number (percentiles); continuous variables are presented as median (interquartile range) unless otherwise stated; cumulative incidences are presented as incidence rates (95% CI).  *Abbreviations:* aGVHD, acute graft‐versus‐host disease; cGVHD, chronic graft‐versus‐host disease; CI, cumulative incidence; CMV, cytomegalovirus; EBV, Epstein–Barr virus; FFS, and failure‐free survival; GFFS, GVHD‐free and failure‐free survival; HAAA, hepatitis‐associated aplastic anaemia; HSCT, haematopoietic stem‐cell transplantation; N, neutrophil; OS, overall survival; PLT, platelet. | |

| 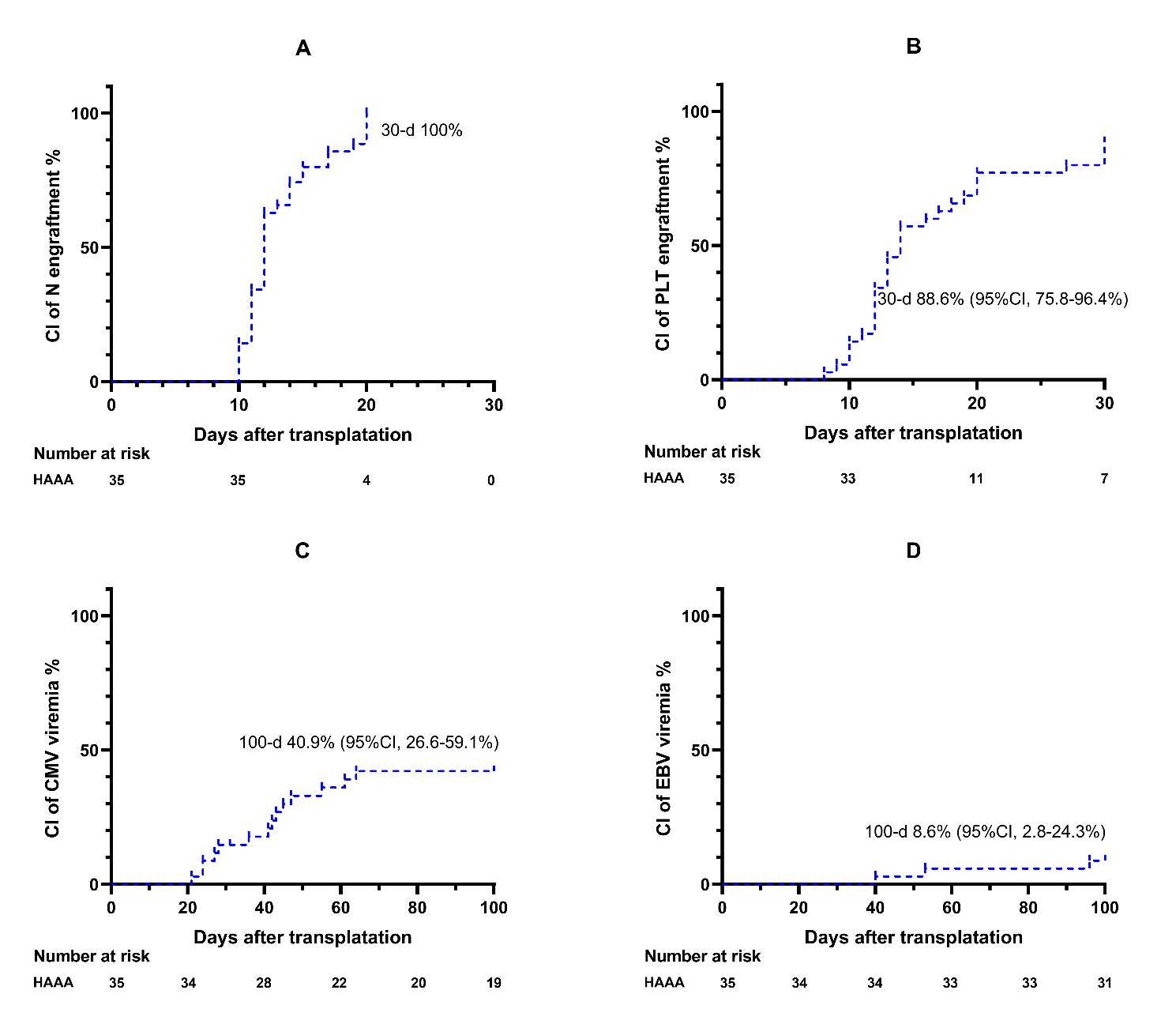 |
| --- |
| Fig.S1 Engraftment and infection in HAAA Patients.  (A) Neutrophil (*N*) engraftment, (B) platelet (PLT) engraftment, (C) cytomegalovirus (CMV) viremia and (D) Epstein-Barr virus (EBV) viremia of patients with HAAA.  *Abbreviations:* CI, cumulative incidence; HAAA, hepatitis-associated aplastic anaemia. |

| 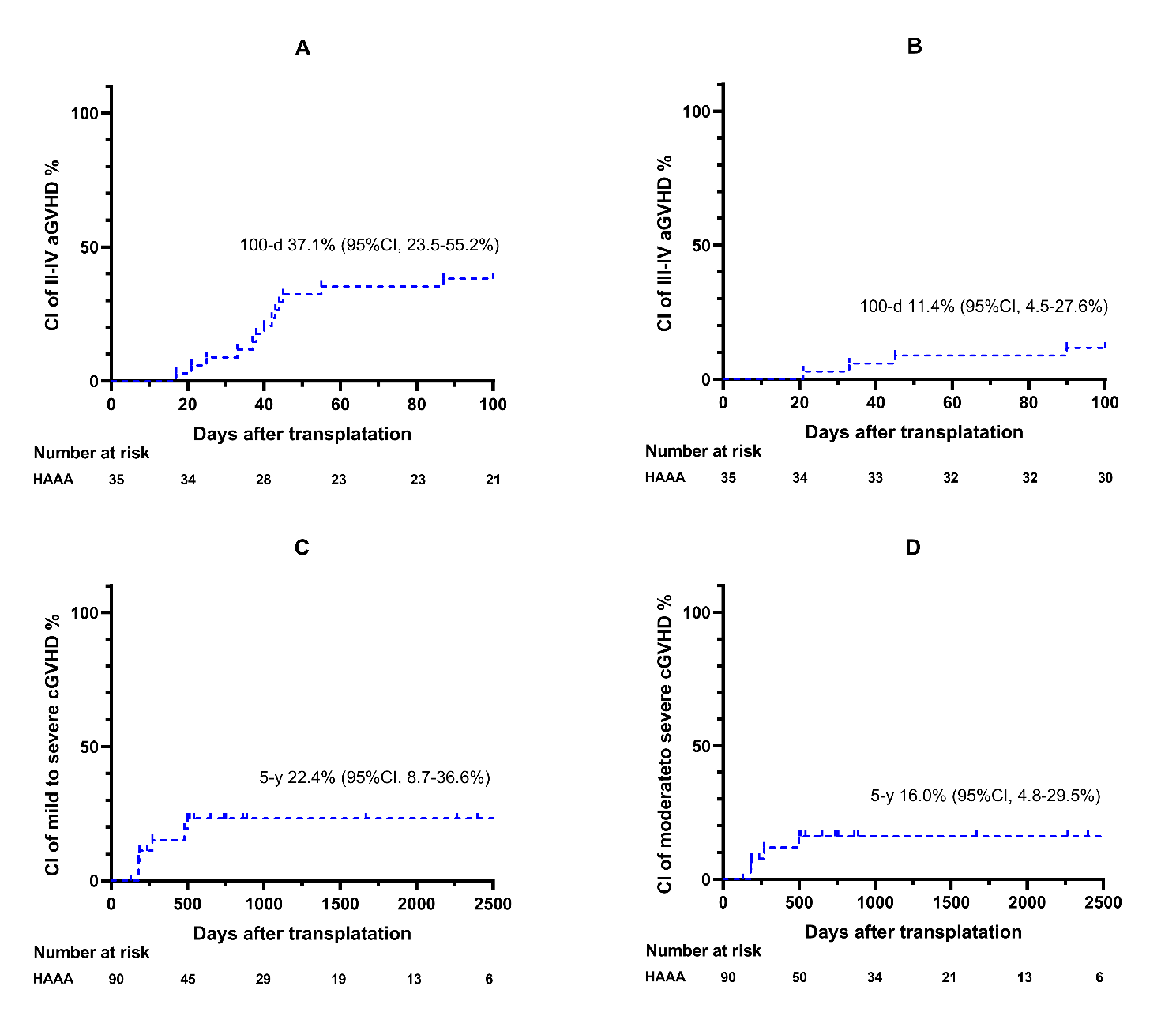 |
| --- |
| Fig.S2 Graft-Versus-Host Disease (GVHD) cumulative incidence in HAAA Patients.  (A) Grade II-IV acute GVHD, (B) grade III-IV acute GVHD, (C) mild to severe chronic GVHD and (D) moderate to severe chronic GVHD are shown.  *Abbreviations:* CI, cumulative incidence; HAAA, hepatitis-associated aplastic anaemia; GVHD, graft-versus-host disease. |
